# CD44 upregulation in chronic liver disease marks the transition to hepatocellular carcinoma and portends poor prognosis

**DOI:** 10.1038/s41416-025-03284-y

**Published:** 2025-12-15

**Authors:** Rui Dong, Akshaya Srikanth, Umesh Tharehalli, Thomas Seufferlein, Reinhold Schirmbeck, André Lechel

**Affiliations:** Department of Internal Medicine I, University Hospital Ulm, Ulm, Germany

**Keywords:** Hepatocellular carcinoma, Cancer models, Prognostic markers, Chronic inflammation

## Abstract

**Background:**

Hepatocellular carcinoma (HCC) often arises from chronic liver disease, but early biomarkers of malignant transformation are lacking. CD44, a transmembrane glycoprotein with multiple isoforms, has been implicated in cancer progression and immune modulation.

**Methods:**

We analysed CD44 expression in mouse models of chronic and acute liver injury and assessed its clinical relevance in human HCC using bulk and single-cell transcriptomic datasets.

**Results:**

CD44 and its isoforms v6 and v10 were progressively upregulated in chronic liver injury, peaking in HCC. CD44-positive hepatocytes increased with fibrosis severity and were abundant in murine liver tumours. In human HCC, CD44 expression was significantly elevated compared to non-tumorous liver and was associated with reduced overall survival. CD44^high^ tumours showed enrichment in oncogenic signalling pathways and greater infiltration of immunosuppressive cells, including M2 macrophages and Th2 cells. Single-cell RNA-seq confirmed CD44 expression in both tumour and immune cells, linking it to a protumor immune microenvironment.

**Conclusions:**

CD44 is a promising early biomarker of hepatocarcinogenesis and a potential therapeutic target. Its expression reflects disease progression from fibrosis to cancer and is associated with poor prognosis and immune evasion in HCC.

## Background

Primary liver cancer is the sixth most frequently diagnosed cancer and the third leading cause of cancer-related deaths globally [1]. Hepatocellular carcinoma (HCC) accounts for approximately 75–85% of primary liver cancers, while intrahepatic cholangiocarcinoma (iCCA) constitutes about 10–15% [2]. In most patients, HCC develops on a background of chronic liver disease [3]. The predominant causes of chronic liver disease are persistent hepatitis B virus (HBV) or hepatitis C virus (HCV) infection, which together contribute to 21–55% of HCC cases worldwide [4]. Other major risk factors include alcohol abuse (leading to alcoholic steatohepatitis, ASH) and the increasingly prevalent metabolic dysfunction-associated fatty liver disease (MAFLD) and steatohepatitis (MASH) [1, 5]. In addition, cholangiocarcinoma (CCA) has its own risk factors such as liver fluke infestation, metabolic conditions (obesity, diabetes, MAFLD), heavy alcohol use, and viral hepatitis [6]. Despite regular surveillance protocols (imaging, clinical assessments, and biochemical parameters), many patients with chronic liver disease are first diagnosed with advanced liver cancer [7].

CD44 is a cell-surface transmembrane glycoprotein expressed by many cell types, playing crucial roles in cell proliferation, adhesion, and migration [8]. CD44 is not a single molecule; rather, it exists in multiple isoforms generated by alternative splicing of ten variable exons in its extracellular domain. The standard isoform (CD44s) contains no variant exon inserts, while variant isoforms (CD44v) include one or more variable exons. CD44s is predominantly expressed in hematopoietic, immune, and epithelial cells, where it regulates immune cell migration and inflammatory responses, as well as maintaining tissue homoeostasis. In contrast, CD44v isoforms show tissue- and disease-specific expression, being markedly upregulated in various cancers (e.g., breast, colon, prostate, ovarian, gastric, liver) and in chronic inflammatory conditions like ulcerative colitis and rheumatoid arthritis [9–12]. In many malignancies, elevated CD44 expression correlates with aggressive tumour behaviour and poor prognosis [13]. Tumour cells overexpressing CD44 often display cancer stem cell traits such as enhanced self-renewal, high tumour-initiating potential, epithelial-mesenchymal transition (EMT) capacity, and chemotherapy resistance [14, 15]. Moreover, CD44 can promote immune evasion by modulating immune cell infiltration in the tumour microenvironment [16]. CD44 also functions as a co-receptor for growth factor receptors (e.g., EGFR and c-Met), activating downstream pathways like NF-κB and STAT3 [17]. Notably, specific CD44 variants impart distinct functions: for example, CD44v6 promotes tumour invasion and metastasis, while CD44v3 enhances cell proliferation and tumour growth by binding heparin and growth factors. These features underscore the biological significance of CD44 isoforms in cancer development and progression, highlighting their potential as diagnostic markers and therapeutic targets [18, 19].

In this study, we evaluated CD44 expression profiles in multiple mouse models of liver disease and regeneration, and examined the prognostic significance of CD44 in human HCC. We demonstrate that high CD44 expression correlates with reduced overall survival in HCC patients. We also show that CD44 (particularly certain splice variants) is upregulated in chronic liver injury in mice, suggesting a role in driving disease progression. Our findings indicate that CD44 is pivotal in the pathogenesis of liver cancer, and that it may serve as both a biomarker of risk in chronic liver disease and a potential therapeutic target to interrupt the progression to malignancy.

## Materials and methods

### Mice

In this study, we utilised six mouse models to investigate the expression of CD44 in acute and chronic liver injury and regeneration. Chronic liver disease models included HBsAg transgenic mice, A1AT/HCV transgenic mice, CCl_4_-treated mice, and Ikk2^ca^ transgenic mice; an acute liver injury model utilised Rbpj conditional knockout mice; and a liver regeneration model was partial hepatectomy.

1. *HBsAg model (74 weeks old mice):* transgenic mice expressing the hepatitis B virus surface antigen (HBsAg) under the liver-specific albumin promoter (C57BL/6J-Tg (Alb1HBV) 44Bri/J) develop chronic liver disease due to accumulation of HBsAg within hepatocytes, leading to endoplasmic reticulum stress and chronic injury [20]. This injury triggers inflammation, regenerative hyperplasia, transcriptional dysregulation, and aneuploidy.
2. *A1AT/HCV model (75 weeks old mice):* transgenic mice carrying the entire HCV open reading frame inserted into the α1-antitrypsin (A1AT) gene exhibit HCV mRNA expression localised to perivascular regions of the liver. These mice develop marked hepatic steatosis with minimal necrosis, along with a consistent T-cell infiltrate and slight hepatocyte proliferation [21].
3. *CCl*_*4*_ *model (32 weeks old mice):* Carbon tetrachloride (CCl_4_) was administered to male C57BL/6 J mice (0.5 ml/kg intraperitoneally, diluted 1:3 in olive oil) twice weekly for 16 weeks to induce chronic liver injury, fibrosis, and eventually liver tumour formation. CCl_4_ is metabolised by cytochrome P450 enzymes to a trichloromethyl radical that damages cellular macromolecules and promotes fibrosis and mutagenesis.
4. *Ikk2*^*ca*^ *model (65 weeks old mice):* Inducible transgenic mice harbouring a constitutively active IKK2 (IKK2^ca^) under a liver-specific tetracycline-inducible system (Tet-off system) (generated by crossing IKK2^ca^ mice with LAP-tTA mice) were used to chronically activate NF-κB signalling [22]. Doxycycline (0.1 g/L in drinking water) was administered to pregnant females to suppress IKK2^ca^ expression in utero; postnatal withdrawal of doxycycline triggered sustained NF-κB activation, inflammatory cell recruitment, hepatocyte proliferation, and liver fibrosis.
5. *Rbpj conditional knockout model (4 weeks old mice):* Liver-specific deletion of the Notch pathway regulator Rbpj was achieved by crossing Rbpj^flox/flox^ mice with AlfpCre transgenic mice (Cre recombinase is expressed under the mouse albumin enhancer and promoter, as well as the mouse alpha-fetoprotein enhancers). Rbpj ablation causes severe cholestasis due to impaired intrahepatic bile duct maturation, leading to extensive hepatocellular necrosis [23].
6. *Partial hepatectomy model (8 weeks old mice):* Male C57BL/6 J mice were subjected to ~70% partial hepatectomy (removal of the left lateral, left median, and right median lobes) under anaesthesia, as described previously [24, 25]. Mice were sacrificed 48 h post-surgery to assess early regenerative responses.

Additionally, HCC tumours were obtained from CCl_4_-treated liver-specific Trp53 knockout mice (Alb-Cre, Trp53^−/−^) *(32 weeks old mice)* [26, 27] following the same CCl_4_ regimen. All transgenic lines were on a C57BL/6 J background and maintained in specific pathogen-free conditions. For each model, five male mice per group were used.

### Histology and immunohistochemistry

Liver tissues were fixed in 4% paraformaldehyde, paraffin-embedded, and sectioned (4–5 μm). Sections were stained with hematoxylin and eosin (H&E) for general histopathology and with Picro-Sirius Red (Direct Red 80/Fast Green) to evaluate fibrosis. For immunohistochemistry, sections underwent deparaffinization, rehydration, and heat-mediated antigen retrieval (EDTA buffer, pH 8.0). Endogenous peroxidase and biotin were blocked before overnight incubation at 4 °C with primary antibodies against CD44 (standard form; #MCA4703, BIO-RAD, 1:100) and CD44v6 (#BMS145, ThermoFisher, 1:300), as well as CD45R/B220 (B cells; #550286, BD Biosciences, 1:100) and CD3ε (T cells; #MA5-14524, Invitrogen, 1:100). Bound antibodies were detected using biotinylated secondary antibodies and a VECTASTAIN ABC peroxidase kit with NovaRED substrate, followed by hematoxylin counterstaining. Integrated optical density (IOD) was measured in five randomly selected fields per section using ImageJ.

### Immunofluorescence co-staining

Liver tissues were fixed in 4% paraformaldehyde, paraffin-embedded, and sectioned at 4 μm. Sections were deparaffinized, rehydrated, and subjected to heat-mediated antigen retrieval using Antigen Unmasking Solution, Citrate-Based (#H-3300-250, VECTOR). After blocking with 10% normal goat or rabbit serum containing 3% BSA in TBST, sections were incubated overnight at 4 °C with three sets of primary antibodies for co-staining: CD44 (#37259, Cell Signaling, 1:200) together with FOXP3 (#14-4771-80, ThermoFisher, 1:200), CD44 (IM7, #14-0441-82, ThermoFisher, 1:400) together with F4/80 (#70076, Cell Signaling, 1:200) and CD44 (#37259, Cell Signaling, 1:200) together with CD206 (#PA5-46994, ThermoFisher, 1:200). The following day, sections were incubated for 1 h at room temperature with a mixture of secondary antibodies, including Cy5-conjugated Goat anti-Rabbit (#81-6116, Invitrogen, 1:200) together with Cy3-conjugated Goat anti-Rat (#A10522, Invitrogen, 1:200), and Cy5-conjugated Goat anti-Rabbit (#81-6116, Invitrogen, 1:200) together with Cy3-conjugated Rabbit anti-Goat (#AP106C, Merck, 1:200). Nuclei were counterstained with ProLong™ Gold Antifade Mountant with DAPI (#P36931, ThermoFisher), and stained sections were examined using a fluorescence microscope.

### Real-time quantitative RT-PCR

Total RNA was isolated from liver tissue using RNeasy kits (QIAGEN) and reverse transcribed into cDNA (Promega GoScript). Quantitative PCR was performed (Applied Biosystems QuantStudio 3) with SYBR Green chemistry to measure expression of CD44 isoforms (Table 1). Gene expression levels were normalised to RNA polymerase II and calculated using the 2^-ΔΔCt^ method.

**Table 1:** Primer sequences.

Gene Symbol	Forward Sequence (5’-3’)	Reverse Sequence (5’-3’)
CD44s	GTCAGCAGCGGCTCCACCATCGAGA	CGCACTTGAGTGTCCAGCTAATTC
CD44v3	GTACGGAGTCAAATACCAACCCAAC	CATCATCATCAATGCCTGATCCAG
CD44v6	CAACTCCTAATAGTACAGCAGAAGC	CAGTTGTCCCTTCTGTCACA
CD44v7	CTTCGGCCCACAACAACCAT	CTTGCTTTCTGTTTGATGACC
CD44v10	CTAAGAGCGGCGCTAAAGAT	CTGGTAAGGAGCCATCAACA
RNA Pol II	CATCAACCAGGTGGTACAGC	GATTCTGGAACTCAACACTCTCC

Table presents primer sequences which were used for qPCR analysis of CD44 transcripts.

### Bioinformatics and data analysis

Human liver cancer data were obtained from public repositories. CD44 immunohistochemistry results pertaining to HCC and CCA were retrieved, along with data from normal liver and adjacent non-tumorous tissue (ANT) for comparison, all sourced from the Human Protein Atlas (HPA) portal (https://www.proteinatlas.org/ENSG00000026508-CD44/pathology/liver+cancer, accessed on August 2, 2025).

Transcriptome data and relevant clinical information from liver fibrosis tissues, cirrhotic tissues, hepatocellular carcinoma (HCC), and normal liver tissues were downloaded from the GEO database (https://www.ncbi.nlm.nih.gov/geo/, accessed on August 5, 2025). Using R, the gene expression matrices and clinical data from each dataset were integrated, and samples with incomplete clinical information or abnormal probe expression were excluded to ensure data quality. The final analysed datasets included the following: GSE62232 (normal tissues (*n* = 10) and HBV-associated liver tumour tissues (*n* = 11)), GSE62232 (normal tissues (*n* = 10) and HCV-associated liver tumour tissues (*n* = 13)), GSE62232 (F0–F1 stage fibrotic tissues (*n* = 22), F2–F3 stage fibrotic tissues *n* = 14), and F4 stage cirrhotic tissues (*n* = 22)), GSE14520 (normal tissues (*n* = 212) and HBV-associated HCC tissues (*n* = 222)), GSE25097 (normal tissues (*n* = 6) and cirrhotic tissues from HCC patients (*n* = 40)), and GSE49541 (F0–F1 stage fibrotic tissues (*n* = 40) and F3–F4 stage fibrotic tissues (*n* = 32) derived from patients with NAFLD). All gene expression data were normalised and log-transformed using the R package “limma” [28] to meet the requirements for subsequent analysis. To investigate the association between CD44 expression and key fibrosis-related genes (COL1A1, ACTA2, and TGFβ1) in human HCC, we used the TIMER 2.0 database (http://cistrome.org/TIMER) [29].

mRNA expression data and clinical information for 371 HCC tumours and 50 adjacent NTL samples were downloaded from The Cancer Genome Atlas (https://portal.gdc.cancer.gov/projects/TCGA-LIHC, accessed on November 5, 2024). Patients were stratified into a CD44^high^ group (TPM ≥ 20, *n* = 56) and CD44^low^ group (TPM ≤ 16, *n* = 259), excluding intermediate expressors, based on CD44 transcript levels. Overall survival differences between CD44^high^ and CD44^low^ groups were evaluated by Kaplan–Meier analysis (log-rank test). An independent survival analysis was performed using GEPIA2 (http://gepia2.cancer-pku.cn/), which integrates TCGA and GTEx data [30].

Differential gene expression between CD44^high^ and CD44^low^ TCGA HCC samples was analysed using edgeR and DESeq2 [31, 32], identifying genes significantly up- or down-regulated in CD44^high^ tumours (with concordant results by both methods). Enrichment of pathways among differentially expressed genes was assessed by Gene Set Variation Analysis (GSVA) [33] and KEGG pathway analysis via clusterProfiler [34]. To examine CD44 at single-cell resolution, To examine CD44 at single-cell resolution, we analysed single-cell RNA-seq datasets from the TISCH2 database (http://tisch.comp-genomics.org/) [35], including the LIHC_GSE166635 dataset (22,631 cells from two treatment-naïve HCC patients) and the CHOL_GSE138709 dataset (33,990 cells from five treatment-naïve iCCA patients). CD44 expression was evaluated across annotated cell types, including malignant cells and various immune subsets. Immune infiltration in bulk tumours was inferred using the xCell algorithm [36] to estimate relative immune cell abundances from TCGA expression data. Correlations between CD44 expression and specific immune cell signatures (e.g., macrophages, Th2 cells, regulatory T cells) in HCC were explored via TISIDB (http://cis.hku.hk/TISIDB) [37].

### Statistical analysis

Analyses of TCGA human data were conducted in R (v4.3.3). Other statistical analyses were performed using GraphPad Prism 8.0. Data are presented as mean ± standard deviation (SD), with SD representing the variation within each group. Prior to statistical testing, data distribution and variance assumptions were assessed. Group differences between two groups were analysed using Mann–Whitney U tests for non-parametric data or Welch’s t-tests for parametric data with unequal variance. For comparisons involving more than two groups, one-way ANOVA followed by Tukey’s post-hoc test was used for parametric data, and Kruskal-Wallis test followed by Dunn’s post-hoc test was used for non-parametric data. Significance thresholds were *=*p* < 0.05; **=*p* < 0.01; ***=*p* < 0.001; ****=*p* < 0.0001.

## Results

**Fig. 1:**
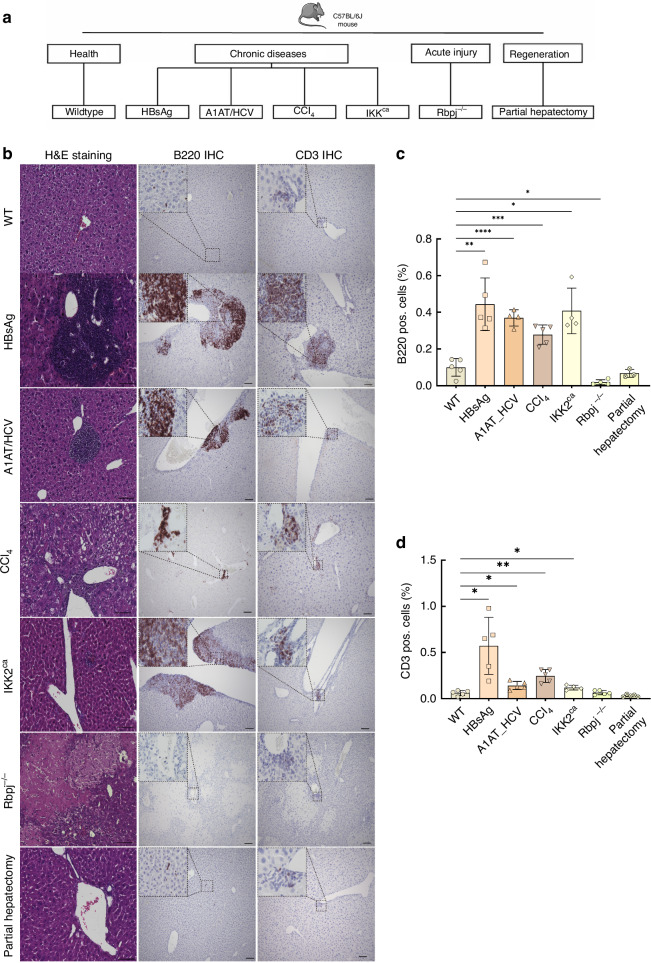
Mouse models. **a** Mouse models of chronic (HBsAg: *n* = 5, A1AT/HCV: *n* = 5, CCl_4_: *n* = 5, IKK2^ca^: *n* = 5, HCC: *n* = 10) and acute liver injury (Rbpj^−/−^: *n* = 5) and liver regeneration (Partial hepatectomy: *n* = 5) used in this study (schematic overview). **b** Histopathology of liver injury models. Representative H&E-stained liver sections from each model (left); **b**, **c** B220 immunohistochemistry (IHC) highlighting B-cell aggregates (brown) (middle); **b**, **d** CD3 IHC highlighting T-cell infiltrates (brown) (right). *=*p* < 0.05; **=*p* < 0.01; ***= *p* < 0.001, ****=*p* < 0.0001.

In the present study, we examined CD44 expression in multiple murine models of liver injury and regeneration (Fig. 1a), and evaluated its clinical significance in human liver cancer.

### Histopathology of chronic, acute, and regenerative liver injury

Chronic liver injury models (HBsAg, A1AT/HCV, CCl_4_, Ikk2^ca^) exhibited expected pathology, including hepatic fibrosis and prominent immune cell infiltration (diffuse inflammation and lymphoid aggregate formation with increased B cells and T cells) (Fig. 1b–d). In contrast, the acute liver injury model (Rbpj^-/-^) showed severe cholestatic damage with extensive hepatocyte necrosis but no lymphoid aggregates, and the liver regeneration model (partial hepatectomy) showed no pathological abnormalities compared to healthy controls.

### CD44v6 and v10 mark hepatocyte commitment to malignant transformation

**Fig. 2:**
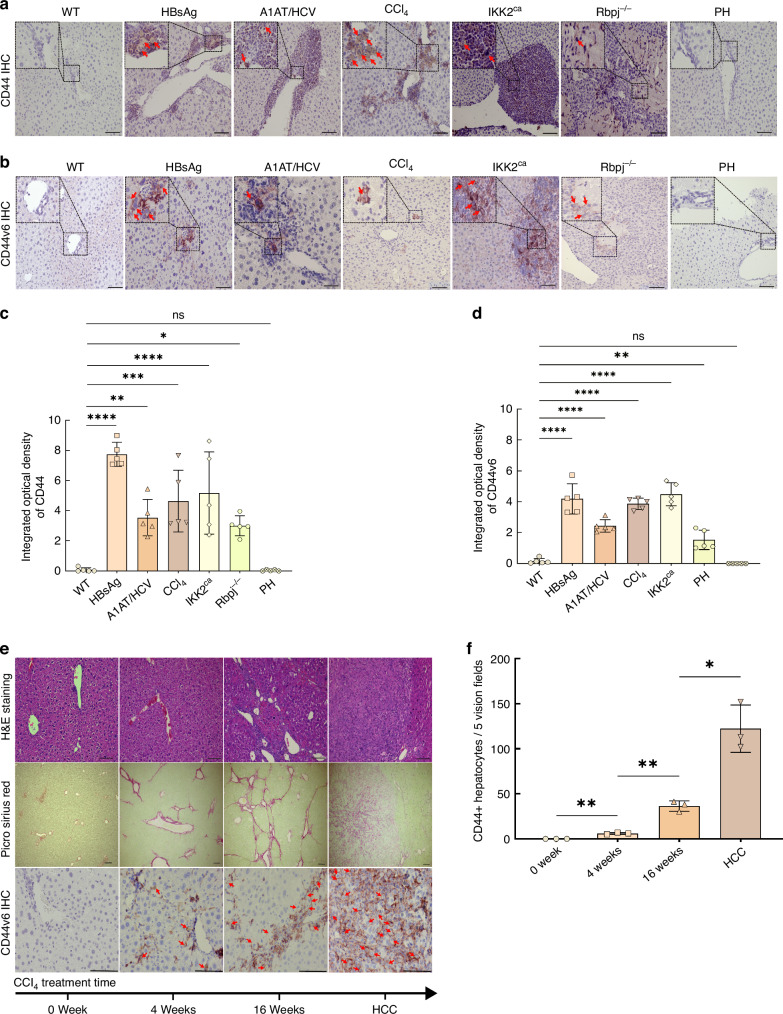
Increase in CD44-positive hepatocytes with fibrosis progression. **a** Representative images of CD44 IHC in injury model livers (arrows indicate CD44-positive hepatocytes). **b** CD44v6 IHC in injury models (arrows indicate CD44v6-positive hepatocytes or cholangiocytes). **c** Quantification of CD44 and (**d**) CD44v6 expression based on integrated optical density (IOD) in the indicated liver injury models. **e** Representative images of liver sections from CCl_4_-treated mice at 4 weeks and 16 weeks, as well as an HCC. H&E staining, Sirius Red staining (fibrosis in red), and CD44 IHC (red arrows point to CD44-positive hepatocytes) are shown. **f** Quantification of CD44-positive hepatocytes in CCl_4_-treated mice over time. *=*p* < 0.05; **=*p* < 0.01.

**Fig. 3:**
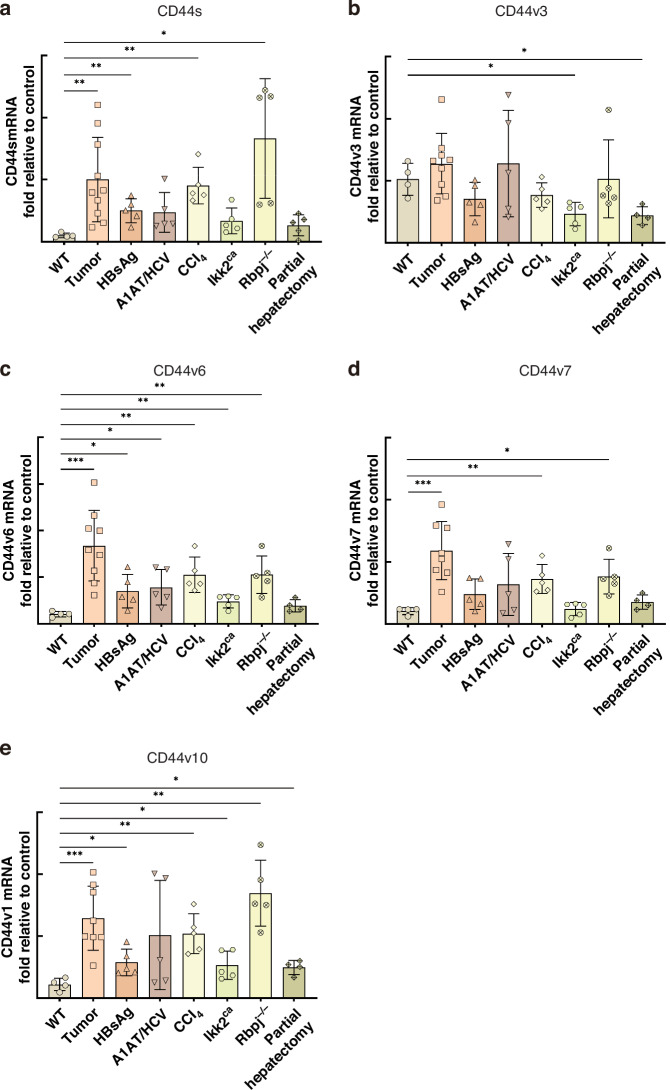
CD44s and CD44v expression in liver injury models. **a** mRNA expression of CD44s (**b**–**e**) and variant isoforms (v3, v6, v7, v10) in liver tissues from different models (n = 4–5 per group). HCC refers to tumours from CCl_4_ treated mice. *=*p* < 0.05; **=*p* < 0.01; ***= *p* < 0.001.

Immunohistochemistry demonstrated that CD44-positive cells are scarce in normal liver and regenerating liver, but become abundant in areas of inflammation in both acute and chronic injury models (Fig. 2a). These CD44-positive cells include infiltrating immune cells and a subset of hepatocytes, often localised near inflammatory foci. Quantitative analysis confirmed a significant increase in CD44-positive staining in acute and chronic injury compared to normal and regenerating liver (Fig. 2c).CD44v6-positive cells were present in all chronic injury models and at low levels in the acute injury model, predominantly in hepatocytes and cholangiocytes within inflamed regions (Fig. 2b), with quantification showing significantly higher CD44v6 staining in chronic injury compared to normal and regenerating liver, and only low levels in acute injury (Fig. 2b); none were detected in regenerating liver. In a time-course of CCl_4_-induced injury, progressive fibrosis and increasing immune cell aggregates were accompanied by a stage-dependent rise in CD44-positive hepatocytes. Livers with advanced fibrosis and especially those with HCC (in the Trp53-deficient CCl_4_ model; AlbCre, Trp53^-/-^ mouse) exhibited markedly higher numbers of CD44-positive hepatocytes than earlier disease stages (Fig. 2e, f). Consistently, CD44s mRNA was markedly upregulated in murine HCC tissues and was also highly expressed in chronically injured livers from the HBsAg and CCl_4_ models; CD44s was moderately elevated in Rbpj^−/−^ (acute injury) livers as well (Fig. 3a). Among variant isoforms, CD44v6, CD44v7, and CD44v10 transcripts were significantly elevated in murine liver tumours (e.g., in HCCs of CCl_4_-treated AlbCre, Trp53^−/−^ mice; Fig. 3c–e) and were also increased in chronic and acute injury livers (particularly CD44v6 and CD44v10). By contrast, CD44v3 expression remained essentially unchanged across models (with a slight decrease in Ikk2^ca^ and regenerating livers; Fig. 3b). These findings suggest that CD44 (especially isoforms v6 and v10) is induced during chronic liver injury and peaks at the malignant stage, suggesting that CD44-positive hepatocytes are associated with the progression from fibrosis to cancer.

### High CD44 expression predicts poor survival in hepatocellular carcinoma

**Fig. 4:**
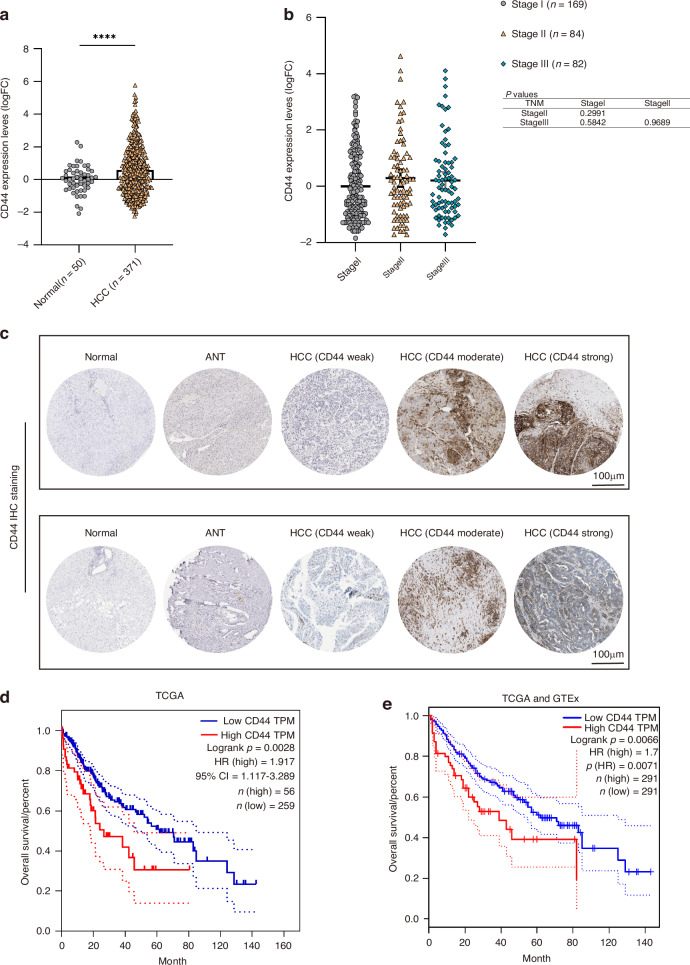
CD44 expression and patient survival in human HCC. **a** CD44 mRNA expression (TPM) in TCGA HCC tumours vs. adjacent normal liver (NTL). **b** CD44 mRNA levels by tumour stage (TCGA HCC). **c** Representative immunohistochemistry images of normal liver, adjacent non-tumorous liver, HCC, and iCCA tissues (images retrieved from the Human Protein Atlas). **d** Kaplan–Meier overall survival curves for TCGA HCC patients with CD44^high^ vs. CD44^low^ tumours. **e** Validation of survival difference in CD44^high^ vs. CD44^low^ groups using GEPIA2 (combined TCGA/GTEx data). ****=*p* < 0.0001.

CD44 mRNA levels were significantly higher in human HCC tumours (*n* = 371) than in adjacent non-tumorous liver tissues (*n* = 50) in the TCGA dataset (*P* < 0.0001; Fig. 4a). Additionally, in a subset of 50 HCC patients with paired tumour and adjacent non-tumorous liver tissues, CD44 mRNA levels were also significantly elevated in tumour tissues compared to their matched normal counterparts (*P* < 0.001; Supplementary Fig. 1). To validate this observation across different liver disease etiologies, we further analysed independent HCC cohorts with HBV or HCV infection backgrounds (GSE62232 and GSE14520). Consistently, CD44 expression was elevated in tumour tissues compared with normal liver in all cohorts (Supplementary Fig. 2a–c), supporting that CD44 upregulation is a general feature of HCC regardless of viral aetiology.

A trend toward higher CD44 expression in Stage II–III tumours compared to Stage I was observed, although not statistically significant (Fig. 4b). However, upon analysis of CD44 expression across different fibrosis stages, a marked increase was observed in advanced fibrosis or cirrhotic tissues. In particular, the GSE62232, GSE49541 and GSE25097 datasets demonstrated higher levels of CD44 in tissues exhibiting advanced fibrosis in comparison to those in the early stages of liver disease or in healthy liver tissues (Supplementary Fig. 3a–c). In addition, within the TCGA HCC cohort, fibrosis-related genes COL1A1, ACTA2, and TGFβ1 exhibited higher levels of expression in the CD44^high^ group (Supplementary Fig. 4a–c), with positive correlations observed between CD44 and those fibrosis-related genes (Supplementary Fig. 4d-f).

Immunohistochemistry from the HPA confirmed that CD44 protein is expressed in both HCC and iCCA tissues, with variable intensity among patients. In contrast, CD44 expression was nearly absent in normal liver and ANT tissues (Fig. 4c). To assess clinical impact, patients were stratified by tumour CD44 expression. Overall survival was significantly worse in the CD44^high^ group (*n* = 56) than in the CD44^low^ group (*n* = 259); median overall survival was ~4.5 years for CD44^high^ vs. ~7.9 years for CD44^low^ tumours (log-rank *P* = 0.0028, HR = 1.917) (Fig. 4d). This association was corroborated using GEPIA2 analysis of a cohort including GTEx data, which also showed shorter survival in patients with high CD44 expression (*P* = 0.0066, HR ≈ 1.7; Fig. 4e). Thus, elevated CD44 expression is linked to poorer prognosis in HCC patients.

### CD44^high^ tumours exhibit oncogenic signatures and immune suppression

**Fig. 5:**
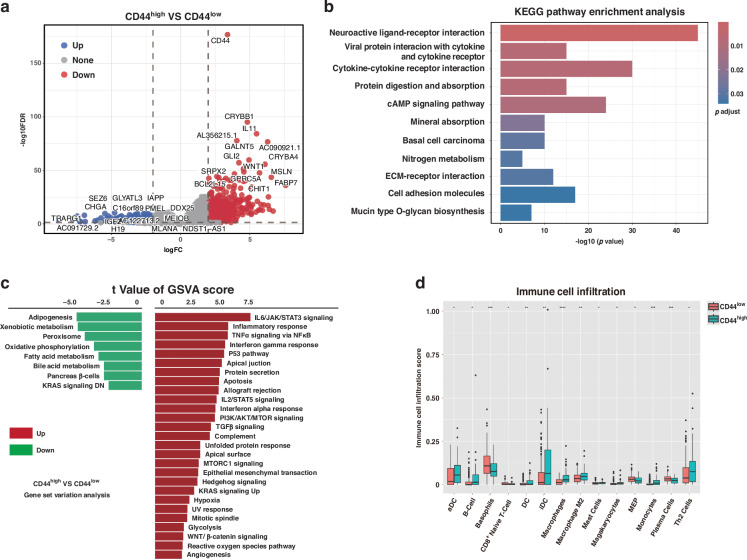
Pathway enrichment and immune profiles of CD44^high^ HCCs. **a** Volcano plot of differentially expressed genes in CD44^high^ vs. CD44^low^ HCC (red dots = upregulated genes; green dots = downregulated genes; selected genes labelled). **b** Top KEGG pathways enriched in CD44^high^ tumours (bar graph of -log_10_ *P* values). **c** GSVA enrichment scores for selected oncogenic pathways (IL6/JAK/STAT3, TNFα/NF-κB, EMT, etc.) in CD44^high^ vs. CD44^low^ tumours. **d** Immune cell fraction comparison (xCell analysis) showing higher M2 macrophage and Th2 cell signatures in CD44^high^ tumours. *=*p* < 0.05; **=*p* < 0.01; ***= *p* < 0.001, ****=*p* < 0.0001.

To explore why CD44^high^ tumours have worse outcomes, we compared their gene expression profiles to CD44^low^ tumours. We identified 504 genes significantly upregulated and 223 genes downregulated in CD44^high^ HCCs (Fig. 5a). Pathway analysis revealed that CD44^high^ tumours were enriched in multiple oncogenic and inflammatory pathways, including cytokine-cytokine receptor interactions, cAMP signalling, ECM-receptor interaction, and key cancer-related signalling cascades (IL6/JAK/STAT3, TNFα/NF-κB, and PI3K/AKT/mTOR) (Fig. 5b, c). These pathways contribute to tumour cell proliferation, survival, metastasis, and immune evasion. Immune cell deconvolution using xCell further showed that CD44^high^ tumours harboured a significantly higher abundance of M2-polarised macrophages (*P* < 0.001) and Th2 cells (*P* < 0.05) compared to CD44^low^ tumours (Fig. 5d). Cytokines (IL-4, IL-13) secreted by Th2 cells have been shown to drive macrophage polarisation towards the pro-tumour M2 phenotype. Furthermore, within the tumour microenvironment, M2 macrophages secrete growth factors and cytokines (e.g., CCL2, CXCL8) that promote tumour growth and suppress anti-tumour immunity.

### Single-cell analysis links CD44 to malignant and immunosuppressive cell populations

**Fig. 6:**
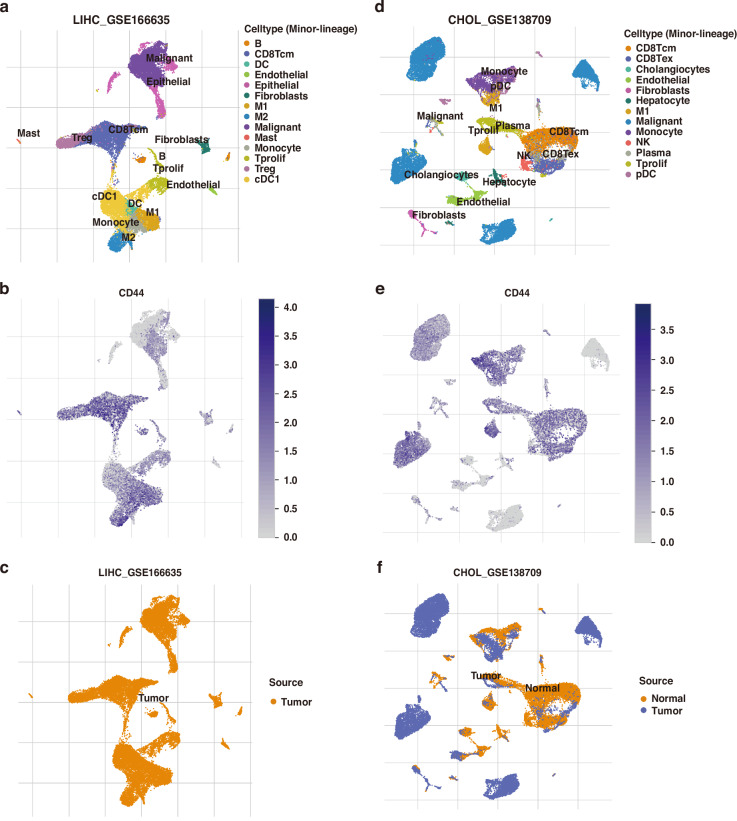
Single-cell analysis of CD44 in liver cancer. UMAP plots of HCC (**a**) and iCCA (**d**) tumours derived from the single-cell RNA-seq datasets GSE166635 (HCC) and GSE138709 (iCCA), with major cell populations annotated (macrophages, T cells, and malignant cells). Feature plots showing CD44 expression levels across specific cell populations in HCC (**b**) and iCCA (**e**) samples (blue = high expression, grey = low expression). UMAP clustering of normal tissues (blue) versus tumour cells (orange) in an HCC sample (**c**) and an iCCA sample (**f**).

Consistent with these findings, single-cell RNA-seq data (TISCH2) for HCC and iCCA revealed high CD44 expression not only in malignant epithelial cells but also in tumour-associated macrophages, T cells, and monocytes (Fig. 6). Notably, HCC tissues with high CD44 expression showed enriched infiltrates of immunosuppressive cell types such as M2 macrophages and regulatory T cells (Tregs). CD44 expression levels also positively correlated with markers of macrophages, Tregs, and Th2 cells in HCC (Supplementary Fig. 8a–c). Collectively, these data suggest that CD44 overexpression in HCC is linked to a pro-tumour immune microenvironment characterised by abundant immunosuppressive infiltrates. This hostile immune milieu, alongside activation of oncogenic pathways, likely contributes to the poor outcomes observed in patients with CD44^high^ tumours.

To further validate these findings in vivo, we performed immunofluorescence co-staining of CD44 with F4/80 (macrophage), CD206 (M2-type macrophage), and Foxp3 (Treg) in liver tissues from WT, Rbpj^**–/–**^, PH, and chronic liver injury models (HBsAg, HCV, CCl₄, and IKK2^**ca**^). Compared with the WT, Rbpj^**–/–**^, and PH groups, the chronic liver injury models exhibited a marked increase in CD44-positive cells, which were predominantly localised within immune cell aggregates (Supplementary Fig. 5). In addition, the presence of CD44-positive hepatocytes was detected in these regions (Supplementary Fig. 5, 7).

F4/80^**+**^ cells were detected across all groups. In WT, Rbpj^**⁻/⁻**^, and PH mice, they mainly represented liver-resident Kupffer cells with low CD44 expression. By contrast, in the chronic injury models, abundant CD44^**+**^ F4/80^**+**^ cells were present, some of which were located within immune cell clusters and often found in close proximity to CD44-positive hepatocytes. In addition, recruited F4/80^**+**^ peripheral monocytes within the immune aggregates also displayed high levels of CD44 expression (Supplementary Fig. 5).

Analysis of macrophage subsets further revealed that M2-type macrophages were markedly increased in chronic liver injury models, predominantly distributed around immune aggregates, and exhibited strong CD44 expression (Supplementary Fig. 6). Notably, Foxp3^**+**^ Tregs were exclusively observed in chronic liver injury models, confined to the immune aggregates but absent in other regions of the hepatic parenchyma. The majority of Foxp3⁺ Tregs also showed robust CD44 positivity (Supplementary Fig. 7).

Collectively, these results suggest that during chronic liver injury, CD44 is not only associated with hepatocytes but also closely linked to immunosuppressive immune cell subsets, consistent with our observations in human HCC.

## Discussion

Liver cancer is marked by extensive heterogeneity in its genetic and molecular alterations, which makes it challenging to find one target that could treat all cases effectively. There is significant interest in identifying biomarkers that are present on tumour cells and can be detected early, during the onset of hepatocarcinogenesis. In this study, we conducted a comprehensive analysis of CD44, a critical cell surface glycoprotein involved in cell-cell interactions, adhesion, migration, and signalling. Consistent with its known role as part of a cancer stem cell signature in multiple tumour types [11], we observed increased CD44 (total) and CD44 variant expression in liver carcinoma compared to non-tumour liver, with a slight increase corresponding to more advanced tumour stage and a clear association with reduced survival probability. Previous studies have reported CD44 as a prognostic marker in HCC, with its subcellular localisation influencing clinical outcomes. Membranous CD44 has been associated with tumour growth, invasion, metastasis, and therapy resistance, correlating with worse survival, increased intrahepatic metastasis, and recurrence. This effect is particularly pronounced when membranous CD44 is co-expressed with wild-type or variant EGFR. In contrast, cytoplasmic CD44, reflecting internalisation, cleavage, or impaired trafficking with reduced surface signalling, has been linked to slower tumour progression and better survival, representing a less aggressive or quiescent phenotype [38]. CD44 has also been proposed as a potential early detection marker for liver cancer, especially in combination with other biomarkers such as TIPRL, LC3, or CD133 [12]. Single-cell and spatial transcriptomic studies of HCC have recently identified CD44 as part of a cancer stem cell profile in an EMT-related tumour cell subtype [39]. Functionally, knocking out CD44 has been shown to reduce liver cancer formation in a transgenic mouse model: loss of CD44 impaired AKT activation, thereby enabling p53 to trigger cell cycle arrest or apoptosis of damaged hepatocytes, rather than allowing them to bypass p53 surveillance and progress to HCC progenitors [17]. These findings underscore CD44’s role in fostering an environment in which genetically altered hepatocytes can survive and clonally expand into cancer.

Beyond patient data, our use of various mouse models illustrates CD44’s potential role in the transition from chronic liver injury to liver cancer. In chronic injury models, we saw that increased CD44 expression coincided with inflammation, fibrosis, and hepatocellular damage. Immune cells infiltrating injured livers (especially in chronic hepatitis/fibrosis models) often expressed CD44, suggesting that either inflammatory signals induce CD44 on local cells or that CD44 helps recruit/retain immune cells in the liver. As liver disease advanced to HCC (particularly in the fibrogenic CCl_4_ model), CD44-positive hepatocytes became markedly more prevalent. This pattern positions CD44 as a candidate biomarker for monitoring disease progression and the impending onset of malignancy in chronic liver disease. We also observed differential expression of CD44 isoforms: notably high CD44v6 and CD44v10 in diseased and cancerous liver tissues, whereas CD44v3 did not increase. This contrasts with other cancers where CD44v3 can be elevated; for instance, in pancreatic cancer CD44v3 promotes tumour progression [40]. Our findings align with earlier clinical reports: Endo et al. found that in HCC, CD44s (standard) was expressed in ~34% of cases and various CD44v isoforms (v5 in 49%, v6 in 27%, v7/8 in 38%, v10 in 24%) were present, with CD44 positivity correlating with poor tumour differentiation and worse survival [41]. Similarly, high levels of CD44s, CD44v6 and CD44v8-10 were associated with unfavourable prognosis in cholangiocarcinoma patients after curative resection [42].

Through transcriptomic profiling, we identified several pathways that distinguish CD44^high^ HCCs. Many of these (IL6/STAT3, TNFα/NF-κB, PI3K/AKT/mTOR, etc.) are well-known drivers of tumour progression and immune regulation [43, 44]. The enrichment of these pathways in CD44^high^ tumours suggests that CD44 might be part of a network that activates pro-tumour signalling programs. On the immune side, our data indicate that CD44^high^ tumours are characterised by a microenvironment skewed towards Th2 cell and M2 macrophage responses. M2 macrophages and Th2 cells are key mediators of immunosuppression in tumours, aiding cancer cells in evading effective immune surveillance [45–48]. CD44 has been implicated in crosstalk between tumour cells, stromal fibroblasts, and immune cells that enhances tumour progression. For example, a positive feedback loop between tumour-derived osteopontin (SPP1) and CD44 on fibroblasts (involving CCN2/TGFβ-TGFBR1 signalling) can sustain an activated stroma that drives EMT and metastasis; disrupting CCN2 breaks this loop and suppresses EMT and metastasis. Additionally, tumour-secreted SPP1 binding to CD44 on hepatic stellate cells activates PI3K/AKT signalling and converts them into cancer-associated fibroblasts, an interaction that can be blocked by anti-CD44 antibodies [49]. In immunosuppressive HCC subtypes (e.g., AFP-positive tumours), SPP1^**+**^ macrophages accumulate and CD44 is upregulated on both T cells and tumour cells. Inhibiting the SPP1-CD44 axis in such models reduced T-cell exhaustion and, especially when combined with anti-PD-1 immunotherapy, significantly decreased tumour burden [32]. These insights illustrate how CD44 expression on tumour and stromal cells can shape an environment conducive to tumour growth and resistant to immune attack.

Consistent with these mechanistic insights, our immunofluorescence analyses in a murine chronic liver injury model further support a critical role for CD44 in shaping an immunosuppressive microenvironment. We observed an enrichment of CD44^**+**^ M2-type macrophages, as well as the presence of CD44^**+**^ Foxp3^**+**^ regulatory T cells. These findings suggest that CD44 may play an active role in the recruitment and maintenance of immunosuppressive immune subsets, thereby reinforcing local immune suppression and creating a microenvironment conducive to tumour development. These findings indicate that CD44 may act as a bridge linking hepatocyte plasticity and immune suppression during the progression from chronic liver injury to HCC by modulating the immune microenvironment.

Taken together, these findings suggest significant clinical implications. CD44 and its isoforms have been identified as potential early biomarkers in patients with chronic liver disease. For instance, the upregulation of CD44 in hepatocytes from cirrhotic biopsies has the potential to serve as a prognostic indicator, suggesting an elevated risk of progression to HCC. Consequently, this could justify the implementation of an intensified surveillance protocol. Moreover, soluble CD44 variants in serum have been associated with disease activity, suggesting that circulating CD44 could help in identifying patients who are undergoing malignant transformation [50]. High tumoral CD44 expression was also significantly associated with poor overall survival in the present cohort, thereby highlighting its potential as a prognostic indicator. The assessment of CD44 expression by immunohistochemistry in biopsy or resection samples has the potential to facilitate risk stratification, and patients with CD44^high^ tumours may benefit from closer postoperative monitoring or additional adjuvant therapy. Incorporation of CD44 into established prognostic models may further improve predictive accuracy. Finally, CD44 has emerged as a promising therapeutic target. Monoclonal antibodies, CD44-targeted drug delivery systems, and imaging probes are currently being investigated for their potential applications in other types of cancer [51–53]. In the context of HCC, the disruption of CD44-hyaluronan or CD44-SPP1 interactions has emerged as a potential therapeutic strategy to counteract the tumour-promoting effects of the microenvironment. Preclinical studies have suggested that CD44 blockade may lead to a reduction in fibroblast activation and T-cell exhaustion, offering a promising avenue for therapeutic intervention. [49, 54, 55]. These findings suggest a potential for integrating CD44 inhibition with immunotherapy. Collectively, these considerations underscore the translational relevance of CD44 as both a biomarker and a promising therapeutic target in liver cancer.

In summary, our study supports an integral role for CD44 and its isoforms in the progression of chronic liver disease to HCC. CD44 upregulation appears early in the course of chronic liver injury, flagging the ongoing development of a tumour-promoting microenvironment, and remains high in a subset of fully transformed tumours that exhibit stem-like features and immunosuppressive properties. Clinically, CD44 could serve as an early biomarker to identify patients with chronic liver disease at elevated risk for HCC, and as a prognostic indicator in HCC patients (with high CD44 expression identifying those with poorer outcomes). Therapeutically, targeting CD44 or its downstream pathways may offer a strategy to interrupt liver cancer development or to improve the efficacy of immunotherapies by altering the tumour microenvironment.

## Conclusion

CD44 and its splice variants are progressively upregulated during the course of chronic liver disease and reach highest levels in HCC. High CD44 expression in HCC is associated with adverse clinicopathological features and reduced patient survival, in conjunction with an immunosuppressive tumour microenvironment. These findings highlight CD44 as a potential biomarker for early liver cancer detection and as a therapeutic target to impede the progression of chronic liver disease to HCC.

## Supplementary information

Supplemental Figures

## Data Availability

All data generated or analysed during this study are included in this published article and its supplementary information files.

## References

[1] Huang DQ, El-Serag HB, Loomba R. Global epidemiology of NAFLD-related HCC: trends, predictions, risk factors and prevention. Nat Rev Gastro Hepat. 2021;18:223–38. 10.1038/s41575-020-00381-6 PMC8016738 33349658

[2] Michalopoulos GK. Hepatostat: liver regeneration and normal liver tissue maintenance. Hepatology. 2017;65:1384–92. 27997988 10.1002/hep.28988

[3] Ringelhan M, Pfister D, O’Connor T, Pikarsky E, Heikenwalder M. The immunology of hepatocellular carcinoma. Nat Immunol. 2018;19:222–32. 29379119 10.1038/s41590-018-0044-z

[4] Rumgay H, Ferlay J, De Martel C, Georges D, Ibrahim AS, Zheng R, et al. Global, regional and national burden of primary liver cancer by subtype. Eur J Cancer. 2022;161:108–18. 34942552 10.1016/j.ejca.2021.11.023

[5] Devarbhavi H, Asrani SK, Arab JP, Nartey YA, Pose E, Kamath PS. Global burden of liver disease: 2023 update. J Hepatol. 2023;79:516–37. 36990226 10.1016/j.jhep.2023.03.017

[6] Clements O, Eliahoo J, Kim JU, Taylor-Robinson SD, Khan SA. Risk factors for intrahepatic and extrahepatic cholangiocarcinoma: a systematic review and meta-analysis. J Hepatol. 2020;72:95–103. 31536748 10.1016/j.jhep.2019.09.007

[7] Colagrande S, Inghilesi AL, Aburas S, Taliani GG, Nardi C, Marra F. Challenges of advanced hepatocellular carcinoma. World J Gastroenterol. 2016;22:7645. 27678348 10.3748/wjg.v22.i34.7645 PMC5016365

[8] Han J, Lee C, Jung Y. Current evidence and perspectives of cluster of differentiation 44 in the liver’s physiology and pathology. Int J Mol Sci. 2024;25:4749. 38731968 10.3390/ijms25094749 PMC11084344

[9] Jordan AR, Racine RR, Hennig MJP, Lokeshwar VB. The role of CD44 in disease pathophysiology and targeted treatment. Front Immunol [Internet]. [cited 2024 Jul 23];6. Available from: http://journal.frontiersin.org/article/10.3389/fimmu.2015.00182/abstract, 2015. 10.3389/fimmu.2015.00182 PMC4404944 25954275

[10] Osawa Y, Kawai H, Tsunoda T, Komatsu H, Okawara M, Tsutsui Y, et al. Cluster of differentiation 44 promotes liver fibrosis and serves as a biomarker in congestive hepatopathy. Hepatol Commun. 2021;5:1437–47. 34430787 10.1002/hep4.1721 PMC8369942

[11] Razmi M, Ghods R, Vafaei S, Sahlolbei M, Saeednejad Zanjani L, Madjd Z. Clinical and prognostic significances of cancer stem cell markers in gastric cancer patients: a systematic review and meta-analysis. Cancer Cell Int. 2021;21:139. 33639931 10.1186/s12935-021-01840-z PMC7912890

[12] Cirillo N. The hyaluronan/CD44 axis: a double-edged sword in cancer. Int J Mol Sci. 2023;24:15812. 37958796 10.3390/ijms242115812 PMC10649834

[13] Du T, Wu Z, Wu Y, Liu Y, Song Y, Ma L. CD44 is associated with poor prognosis of ccRCC and facilitates ccRCC cell migration and invasion through HAS1/MMP9. Biomedicines. 2023;11:2077. 37509716 10.3390/biomedicines11072077 PMC10377257

[14] Han J, Won M, Kim JH, Jung E, Min K, Jangili P, et al. Cancer stem cell-targeted bio-imaging and chemotherapeutic perspective. Chem Soc Rev. 2020;49:7856–78. 32633291 10.1039/d0cs00379d

[15] Hassn Mesrati M, Syafruddin SE, Mohtar MA, Syahir A. CD44: A Multifunctional mediator of cancer progression. Biomolecules. 2021;11:1850. 34944493 10.3390/biom11121850 PMC8699317

[16] Yang Y, Sun H, Yu H, Wang L, Gao C, Mei H, et al. Tumor-associated-fibrosis and active collagen-CD44 axis characterize a poor-prognosis subtype of gastric cancer and contribute to tumor immunosuppression. J Transl Med. 2025;23:123. 39871345 10.1186/s12967-025-06070-9 PMC11773867

[17] Dhar D, Antonucci L, Nakagawa H, Kim JY, Glitzner E, Caruso S, et al. Liver cancer initiation requires p53 inhibition by CD44-enhanced growth factor signaling. Cancer Cell. 2018;33:1061–.e6. 29894692 10.1016/j.ccell.2018.05.003 PMC6005359

[18] Senbanjo LT, Chellaiah MA. CD44: a multifunctional cell surface adhesion receptor is a regulator of progression and metastasis of cancer cells. Front Cell Dev Biol [Internet]. [cited 2024 Jul 31];5. Available from: http://journal.frontiersin.org/article/10.3389/fcell.2017.00018/full, 2017. 10.3389/fcell.2017.00018 PMC5339222 28326306

[19] Chen C, Zhao S, Karnad A, Freeman JW. The biology and role of CD44 in cancer progression: therapeutic implications. J Hematol Oncol. 2018;11:64. 29747682 10.1186/s13045-018-0605-5 PMC5946470

[20] Chisari FV, Filippi P, Buras J, McLachlan A, Popper H, Pinkert CA, et al. Structural and pathological effects of synthesis of hepatitis B virus large envelope polypeptide in transgenic mice. Proc Natl Acad Sci USA. 1987;84:6909–13. 3477814 10.1073/pnas.84.19.6909 PMC299194

[21] Alonzi T, Agrati C, Costabile B, Cicchini C, Amicone L, Cavallari C, et al. Steatosis and intrahepatic lymphocyte recruitment in hepatitis C virus transgenic mice. J Gen Virol. 2004;85:1509–20. 15166435 10.1099/vir.0.19724-0

[22] Svinarenko M, Katz S-F, Tharehalli U, Mulaw MA, Maier HJ, Sunami Y, et al. An IKK/NF-κB activation/p53 deletion sequence drives liver carcinogenesis and tumor differentiation. Cancers. 2019;11:1410. 31546614 10.3390/cancers11101410 PMC6827060

[23] Tharehalli U, Svinarenko M, Kraus JM, Kühlwein SD, Szekely R, Kiesle U, et al. YAP activation drives liver regeneration after cholestatic damage induced by Rbpj deletion. Int J Mol Sci. 2018;19:3801. 30501048 10.3390/ijms19123801 PMC6321044

[24] Higgins GM, Anderson RM. Experimental pathology of the liver: restoration of the liver of the white rat following partial surgical removal. Arch Pathol. 1931;12:186–202.

[25] Nevzorova Y, Tolba R, Trautwein C, Liedtke C. Partial hepatectomy in mice. Lab Anim. 2015;49:81–8. 25835741 10.1177/0023677215572000

[26] Postic C, Shiota M, Niswender KD, Jetton TL, Chen Y, Moates JM, et al. Dual roles for glucokinase in glucose homeostasis as determined by liver and pancreatic β cell-specific gene knock-outs using Cre recombinase. J Biol Chem. 1999;274:305–15. 9867845 10.1074/jbc.274.1.305

[27] Marino S, Vooijs M, Van Der Gulden H, Jonkers J, Berns A. Induction of medulloblastomas in *p53* -null mutant mice by somatic inactivation of *rb* in the external granular layer cells of the cerebellum. Genes Dev. 2000;14:994–1004. 10783170 PMC316543

[28] Ritchie ME, Phipson B, Wu D, Hu Y, Law CW, Shi W, et al. limma powers differential expression analyses for RNA-sequencing and microarray studies. Nucleic Acids Res. 2015;43:e47. 25605792 10.1093/nar/gkv007 PMC4402510

[29] Li B, Severson E, Pignon J-C, Zhao H, Li T, Novak J, et al. Comprehensive analyses of tumor immunity: implications for cancer immunotherapy. Genome Biol. 2016;17:174. 27549193 10.1186/s13059-016-1028-7 PMC4993001

[30] Tang Z, Kang B, Li C, Chen T, Zhang Z. GEPIA2: an enhanced web server for large-scale expression profiling and interactive analysis. Nucleic Acids Res. 2019;47:W556–60. 31114875 10.1093/nar/gkz430 PMC6602440

[31] Robinson MD, McCarthy DJ, Smyth GK. edgeR: a Bioconductor package for differential expression analysis of digital gene expression data. Bioinformatics. 2010;26:139–40. 19910308 10.1093/bioinformatics/btp616 PMC2796818

[32] Love MI, Huber W, Anders S. Moderated estimation of fold change and dispersion for RNA-seq data with DESeq2. Genome Biol. 2014;15:550. 25516281 10.1186/s13059-014-0550-8 PMC4302049

[33] Hänzelmann S, Castelo R, Guinney J. GSVA: gene set variation analysis for microarray and RNA-Seq data. BMC Bioinf. 2013;14:7. 10.1186/1471-2105-14-7 PMC3618321 23323831

[34] Yu G, Wang L-G, Han Y, He Q-Y. clusterProfiler: an R package for comparing biological themes among gene clusters. OMICS J Integr Biol. 2012;16:284–7. 10.1089/omi.2011.0118 PMC3339379 22455463

[35] Han Y, Wang Y, Dong X, Sun D, Liu Z, Yue J, et al. TISCH2: expanded datasets and new tools for single-cell transcriptome analyses of the tumor microenvironment. Nucleic Acids Res. 2023;51:D1425–31. 36321662 10.1093/nar/gkac959 PMC9825603

[36] Aran D, Hu Z, Butte AJ. xCell: digitally portraying the tissue cellular heterogeneity landscape. Genome Biol. 2017;18:220. 29141660 10.1186/s13059-017-1349-1 PMC5688663

[37] Ru B, Wong CN, Tong Y, Zhong JY, Zhong SSW, Wu WC, et al. TISIDB: an integrated repository portal for tumor–immune system interactions. Wren J, editor. Bioinformatics. 2019;35:4200–2. 10.1093/bioinformatics/btz210 30903160

[38] Sherif O, Khelwatty S, Bagwan I, Seddon A, Dalgleish A, Mudan S, et al. Expression of EGFRvIII and its co‑expression with wild‑type EGFR, or putative cancer stem cell biomarkers CD44 or EpCAM are associated with poorer prognosis in patients with hepatocellular carcinoma. Oncol Rep. 2024;52:172. 39450530 10.3892/or.2024.8831 PMC11526438

[39] Guo D-Z, Zhang X, Zhang S-Q, Zhang S-Y, Zhang X-Y, Yan J-Y, et al. Single-cell tumor heterogeneity landscape of hepatocellular carcinoma: unraveling the pro-metastatic subtype and its interaction loop with fibroblasts. Mol Cancer. 2024;23:157. 39095854 10.1186/s12943-024-02062-3 PMC11295380

[40] Zhu H, Zhou W, Wan Y, Lu J, Ge K, Jia C. CD44V3, an alternatively spliced form of CD44, promotes pancreatic cancer progression. Int J Mol Sci. 2022;23:12061. 36292918 10.3390/ijms232012061 PMC9603666

[41] Endo K, Terada T. Protein expression of CD44 (standard and variant isoforms) in hepatocellular carcinoma: relationships with tumor grade, clinicopathologic parameters, p53 expression, and patient survival. J Hepatol. 2000;32:78–84. 10673070 10.1016/s0168-8278(00)80192-0

[42] Titapun A, Luvira V, Srisuk T, Jareanrat A, Thanasukarn V, Thanee M, et al. High levels of serum IgG for Opisthorchis viverrini and CD44 expression predict worse prognosis for cholangiocarcinoma patients after curative resection. Int J Gen Med. 2021;14:2191–204. 34103974 10.2147/IJGM.S306339 PMC8179826

[43] Johnson DE, O’Keefe RA, Grandis JR. Targeting the IL-6/JAK/STAT3 signalling axis in cancer. Nat Rev Clin Oncol. 2018;15:234–48. 29405201 10.1038/nrclinonc.2018.8 PMC5858971

[44] Su P, Jiang L, Zhang Y, Yu T, Kang W, Liu Y, et al. Crosstalk between tumor-associated macrophages and tumor cells promotes chemoresistance via CXCL5/PI3K/AKT/mTOR pathway in gastric cancer. Cancer Cell Int. 2022;22:290. 36151545 10.1186/s12935-022-02717-5 PMC9508748

[45] Frafjord A, Buer L, Hammarström C, Aamodt H, Woldbæk PR, Brustugun OT, et al. The immune landscape of human primary lung tumors is Th2 skewed. Front Immunol. 2021;12:764596. 34868011 10.3389/fimmu.2021.764596 PMC8637168

[46] Shang Q, Yu X, Sun Q, Li H, Sun C, Liu L. Polysaccharides regulate Th1/Th2 balance: a new strategy for tumor immunotherapy. Biomed Pharmacother. 2024;170:115976. 38043444 10.1016/j.biopha.2023.115976

[47] Wang H, Wang X, Zhang X, Xu W. The promising role of tumor-associated macrophages in the treatment of cancer. Drug Resist Updat. 2024;73:101041. 38198845 10.1016/j.drup.2023.101041

[48] Zhou Y, Qian M, Li J, Ruan L, Wang Y, Cai C, et al. The role of tumor-associated macrophages in lung cancer: from mechanism to small molecule therapy. Biomed Pharmacother. 2024;170:116014. 38134634 10.1016/j.biopha.2023.116014

[49] Tong W, Wang T, Bai Y, Yang X, Han P, Zhu L, et al. Spatial transcriptomics reveals tumor-derived SPP1 induces fibroblast chemotaxis and activation in the hepatocellular carcinoma microenvironment. J Transl Med. 2024;22:840. 39267037 10.1186/s12967-024-05613-w PMC11391636

[50] Molica S, Vitelli G, Levato D, Giannarelli D, Gandolfo GM. Elevated serum levels of soluble CD44 can identify a subgroup of patients with early B-cell chronic lymphocytic leukemia who are at high risk of disease progression. Cancer. 2001;92:713–9. 11550139 10.1002/1097-0142(20010815)92:4<713::aid-cncr1374>3.0.co;2-o

[51] Mohammadi AH, Bagheri F, Baghaei K. Chondroitin sulfate-tocopherol succinate modified exosomes for targeted drug delivery to CD44-positive cancer cells. Int J Biol Macromol. 2024;275:133625. 39084997 10.1016/j.ijbiomac.2024.133625

[52] Li J, Li M, Tian L, Qiu Y, Yu Q, Wang X, et al. Facile strategy by hyaluronic acid functional carbon dot-doxorubicin nanoparticles for CD44 targeted drug delivery and enhanced breast cancer therapy. Int J Pharm. 2020;578:119122. 32035259 10.1016/j.ijpharm.2020.119122

[53] Ejima R, Suzuki H, Tanaka T, Asano T, Kaneko MK, Kato Y. Development of a novel anti-CD44 variant 6 monoclonal antibody C44Mab-9 for multiple applications against colorectal carcinomas. Int J Mol Sci. 2023;24:4007. 36835416 10.3390/ijms24044007 PMC9965047

[54] Li J-H, Wang Y-C, Qin C-D, Yao R-R, Zhang R, Wang Y, et al. Over expression of hyaluronan promotes progression of HCC via CD44-mediated pyruvate kinase M2 nuclear translocation. Am J Cancer Res. 2016;6:509–21. PMC4859677 27186420

[55] Su Z, Zhong Y, He Y, You L, Xin F, Wang L, et al. Bulk- and single cell-RNA sequencing reveal KIF20A as a key driver of hepatocellular carcinoma progression and immune evasion. Front Immunol. 2024;15:1469827. 39555078 10.3389/fimmu.2024.1469827 PMC11563802

