# Supplementary material for: CD44 upregulation in chronic liver disease marks the transition to hepatocellular carcinoma and portends poor prognosis
Source: Br J Cancer. 2025 Dec 15;134(4):555–66. doi: 10.1038/s41416-025-03284-y (PMC12858952; doi:10.1038/s41416-025-03284-y)
Supplement: Supplementary file 1 — Supplemental Figures [file 41416_2025_3284_MOESM1_ESM.docx]

**Supplementary Information**

**CD44 upregulation in chronic liver disease marks the transition to hepatocellular carcinoma and portends poor prognosis**

Rui Dong, Akshaya Srikanth, Umesh Tharehalli, Thomas Seufferlein, Reinhold Schirmbeck, André Lechel

**Table of contents:**

Page 2:

**Supplemental Fig. 1: CD44 expression in adjacent normal and tumor tissues of HCC patients.**

Page 3:

**Supplemental Fig. 2:** **CD44 mRNA expression in HCC patients with different viral backgrounds.**

Page 4:

**Supplemental Fig. 3:** **CD44 mRNA expression in human liver tissues exhibiting varying degrees of fibrosis severity.**

Page 5:

**Supplemental Fig. 4: Correlation between CD44 expression and fibrosis-related genes in patients with hepatocellular carcinoma.**

Page 6:

**Supplemental Fig. 5: CD44 and F4/80 co-immunofluorescence staining in mouse liver disease models.**

Page 7:

**Supplemental Fig. 6:** **CD44 and CD206 co-immunofluorescence staining in mouse liver disease models.**

Page 8:

**Supplemental Fig. 7: CD44 and FoxP3 co-immunofluorescence staining in mouse liver disease models.**

Page 9:

**Supplemental Fig. 8: Correlation between CD44 expression levels and the abundance of immune cell subsets in HCC tissues.**


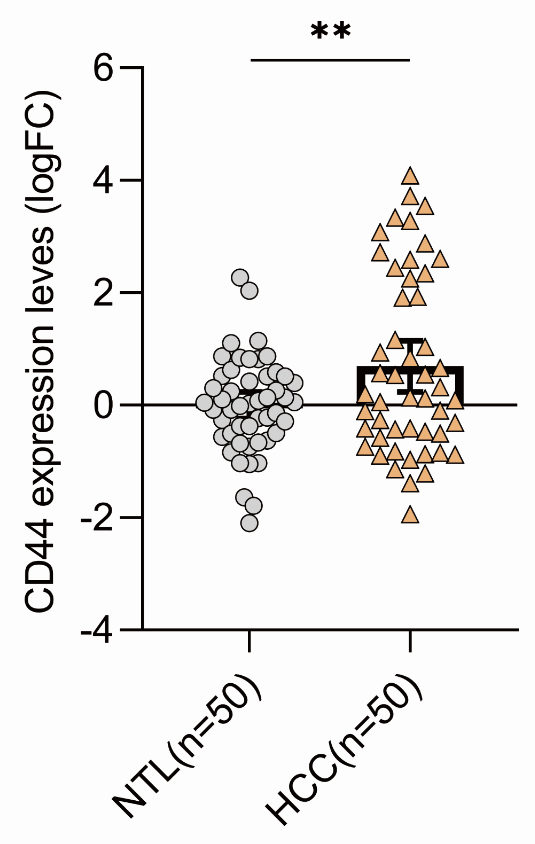


**Supplemental Fig. 1: CD44 expression in adjacent normal and tumor tissues of HCC patients.**

CD44 mRNA expression (TPM) in paired TCGA HCC tumors and adjacent normal liver (NTL) from 50 HCC patients. ***p* < 0.01.

**
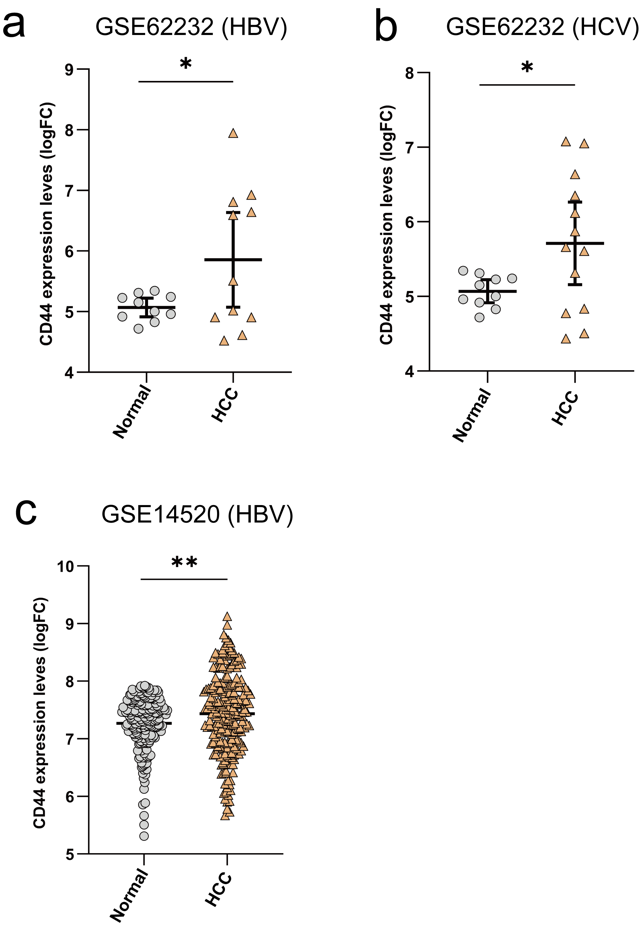
**

**Supplemental Fig. 2:** **CD44 mRNA expression in HCC patients with different viral backgrounds.**

(a) Comparison of HCC tissues from HBV-infected patients (GSE62232) with normal liver tissues. (b) Comparison of HCC tissues from HCV-infected patients (GSE62232) with normal liver tissues. The samples from normal liver tissues shown in (a) and (b) are identical. (c) Comparison of HCC tissues from HBV-infected patients (GSE14520) with normal liver tissues. **p* < 0.05, ***p* < 0.01.


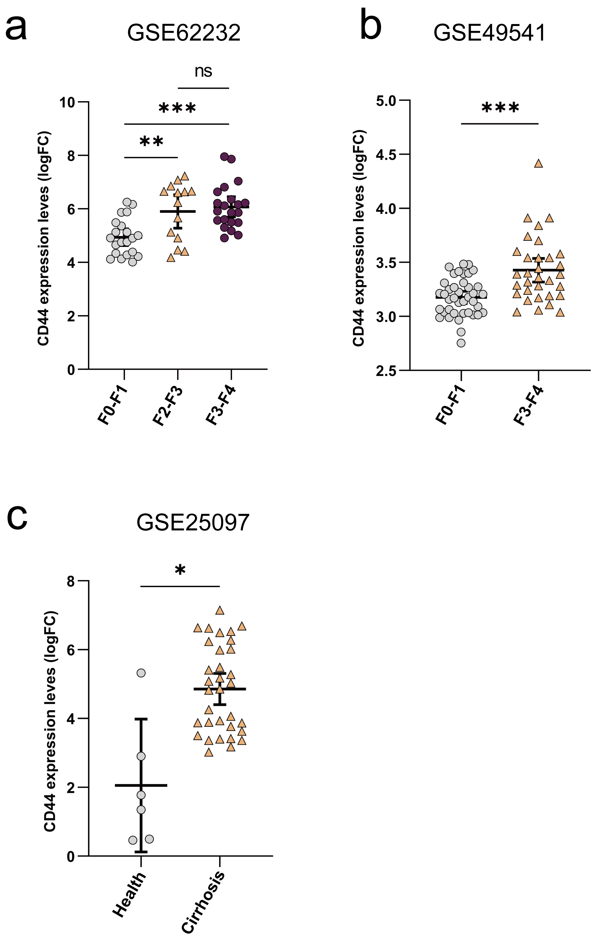


**Supplemental Fig. 3:** **CD44 mRNA expression in human liver tissues exhibiting varying degrees of fibrosis severity.**

(a) CD44 expression in F0-1, F2-3, and F3-4 fibrosis stages from the GSE62232 dataset. (b) CD44 expression in F0-1 versus F3-4 fibrosis stages in patients with NAFLD from the GSE49541 dataset. (c) CD44 expression in cirrhotic versus healthy liver tissues from the GSE25097 dataset. *=*p* < 0.05; ***= *p* < 0.001.


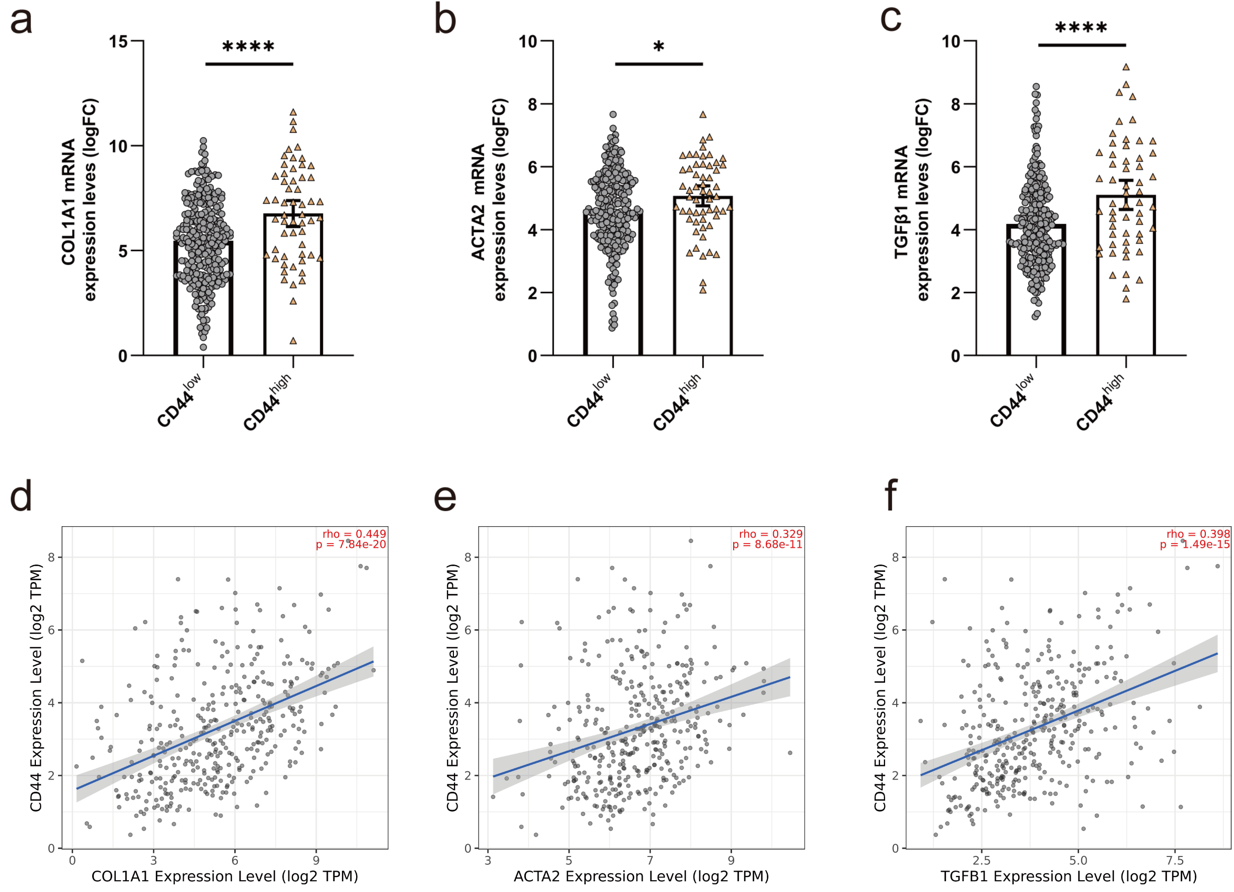


**Supplemental Fig. 4: Correlation between CD44 expression and fibrosis-related genes in patients with hepatocellular carcinoma.**

(a) COL1A1 expression in CD44^high^ and CD44^low^ expressing HCC. (b) ACTA2 expression in CD44^high^ and CD44^low^ HCC. (c) TGFβ1 expression in CD44^high^ and CD44^low^ HCC.

(e) Correlation plot between CD44 and COL1A1 in HCC patients. (f) Correlation plot between CD44 and COL1A1 in HCC patients. (g) Correlation plot between CD44 and COL1A1 in HCC patients. *=*p* < 0.05; ****=*p* < 0.0001.


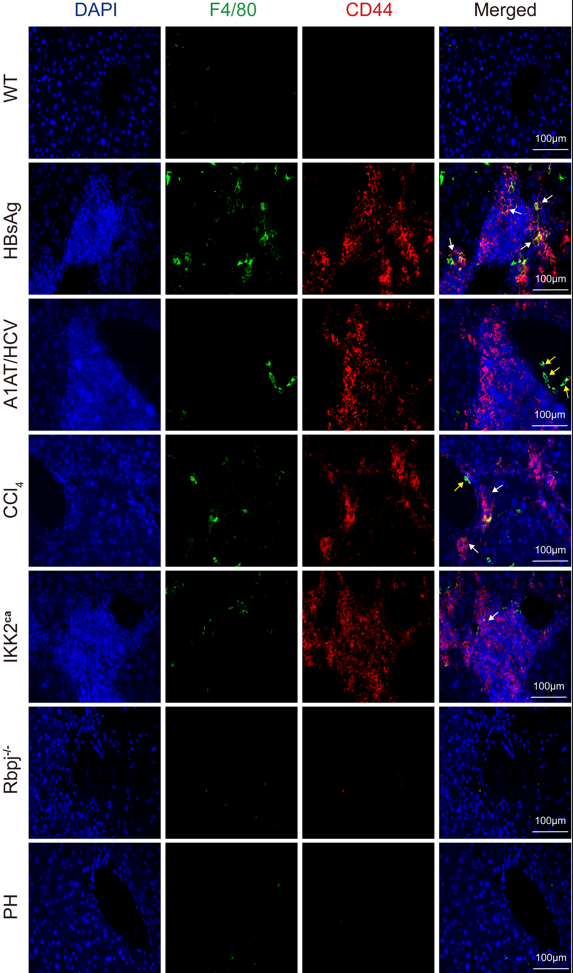


**Supplemental Fig. 5: CD44 and F4/80 co-immunofluorescence staining in mouse liver disease models.**

Representative immunofluorescence staining of CD44 (red) and F4/80 (green) in liver tissues. Nuclei were counterstained with DAPI (blue). White arrows indicate CD44-positive hepatocytes, and yellow arrows indicate infiltrating monocytes recruited from the periphery.


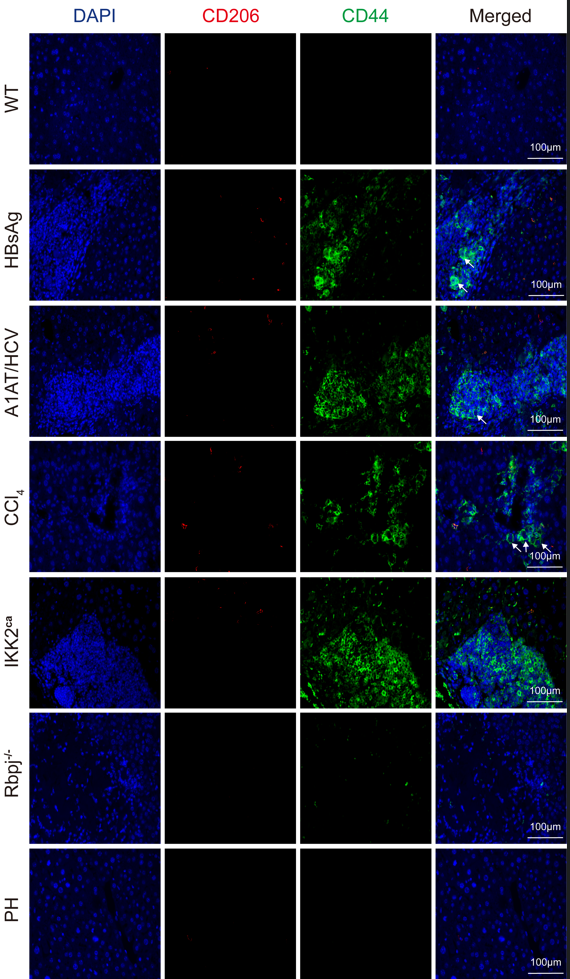


**Supplemental Fig. 6:** **CD44 and CD206 co-immunofluorescence staining in mouse liver disease models.**

Representative immunofluorescence staining of CD44 (green) and CD206 (red) in liver tissues. Nuclei were counterstained with DAPI (blue). White arrows indicate CD44-positive hepatocytes.


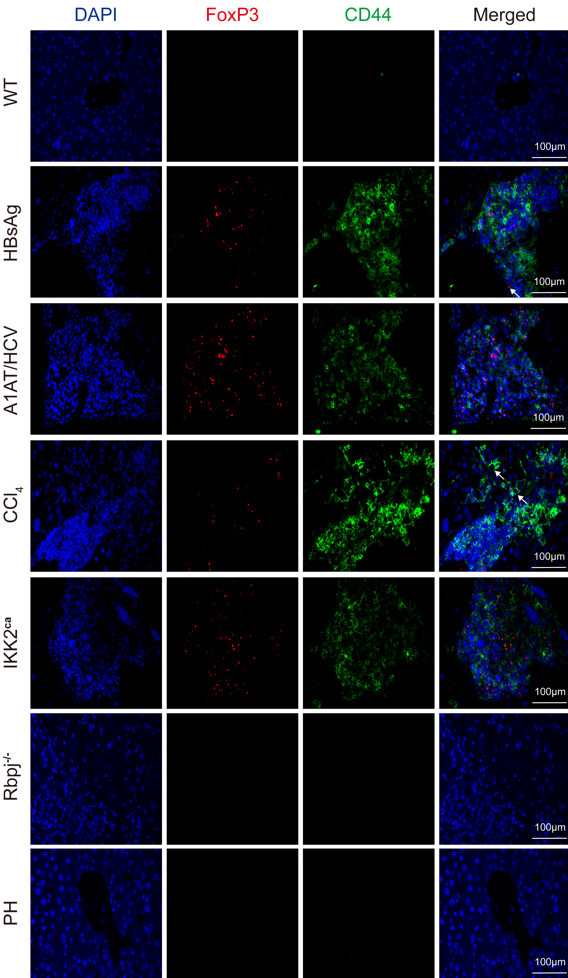


**Supplemental Fig. 7: CD44 and FoxP3 co-immunofluorescence staining in mouse liver disease models.**

Representative immunofluorescence staining of CD44 (green) and Foxp3 (red) in liver tissues. Nuclei were counterstained with DAPI (blue). White arrows indicate CD44-positive hepatocytes.


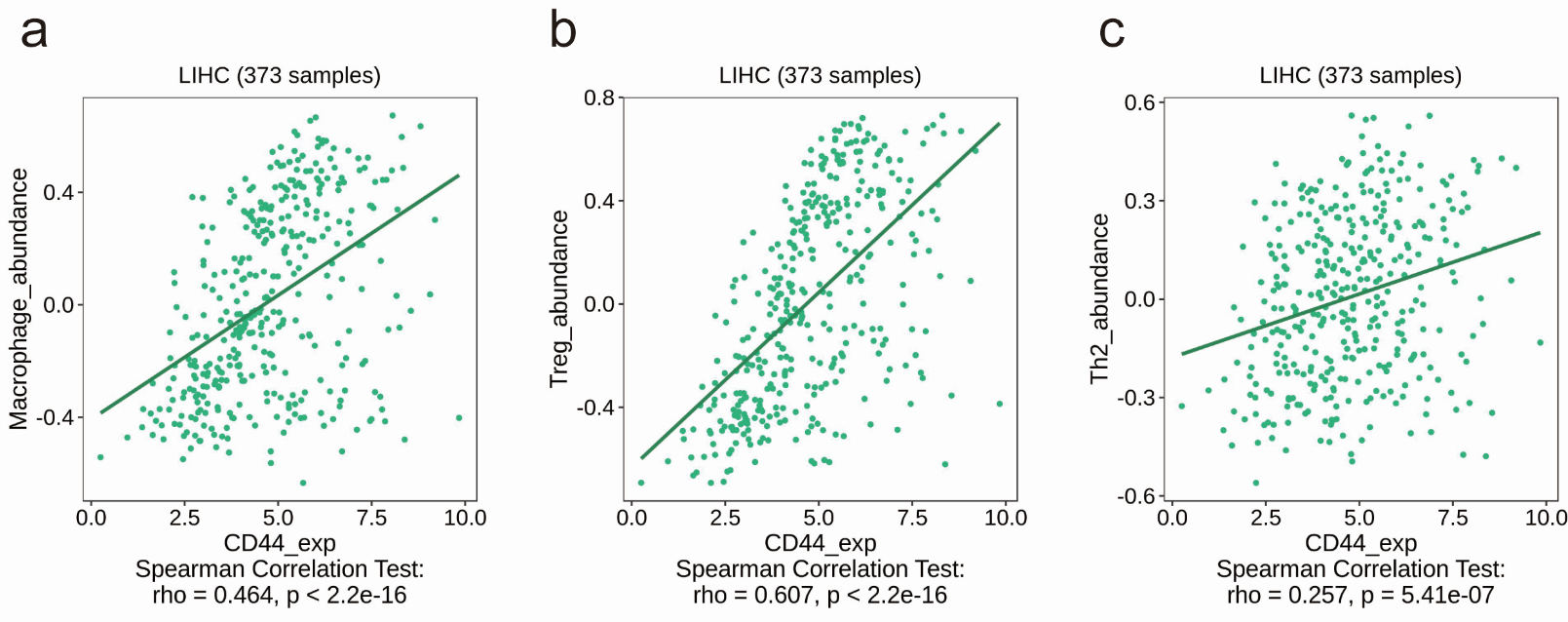


**Supplemental Fig. 8: Correlation between CD44 expression levels and the abundance of immune cell subsets in HCC tissues.**

(a) Correlation plot between CD44 expression and the abundance of macrophages. (b) Correlation plot between CD44 expression and the abundance of Treg cells. (c) Correlation plot between CD44 expression and the abundance of Th2 cells.
